# Barriers and Facilitators to Implementing Acute Pain Services at King Faisal Hospital Rwanda

**DOI:** 10.1155/prm/8360891

**Published:** 2025-03-27

**Authors:** Gaston Nyirigira, Felix Rutayisire, Jackson Kwizera Ndekezi, Rulinda Kwizera, Kara L. Neil

**Affiliations:** ^1^Department of Anesthesia and Critical Care, King Faisal Hospital Rwanda, Kigali, Rwanda; ^2^Department of Academic Affairs and Research, Africa Health Sciences University, Kigali, Rwanda; ^3^Division of Education, Training, and Research, King Faisal Hospital Rwanda, Kigali, Rwanda

**Keywords:** acute pain service, barriers, facilitators, Rwanda

## Abstract

**Background:** As a critical component of clinical care, every patient should have access to acute pain service (APS). Despite significant progress in its development, acute pain is under or inadequately treated, particularly in African countries. In addition, acute pain treatment and management has received insufficient clinical attention, resulting in inadequacies in postoperative pain relief, which has continued to be a significant challenge.

**Aims:** This study aims to assess the knowledge, perceptions, and experiences of healthcare professionals about APS delivery at King Faisal Hospital Rwanda (KFH).

**Methods:** Nine semistructured focus group discussions (FGDs) were conducted from April to May 2023. Participants were selected via random stratified sampling, and FGDs were conducted in internal medicine, anesthesia and the operating theater, obstetrics and gynecology, the intensive care unit, pediatrics, accident and emergency, medical doctors, physiotherapy, and the surgical ward departments at KFH.

**Results:** Participants highlighted four key areas that can serve as either barriers or facilitators to implementing APS at KFH. These include healthcare provider skills and training; the development and implementation of standardized protocols; establishing a dedicated interdisciplinary APS team; and patient awareness and education.

**Conclusions:** Having institutional systems in place, including standardized protocols, a dedicated team, and regular training opportunities, may help strengthen APS. Patient education and ensuring patients know their care options is another facilitator to improving APS.

## 1. Introduction

Clinical research posits that acute pain is caused by either a disease or injury and is associated with muscle contraction and related nervous system activation, resulting in intense pain [1]. As a result, acute pain is a threat to other organ systems, which can lead to significant complications and the development of chronic pain. Additionally, while it is regularly administered to surgical patients, acute pain management could also be provided to nonsurgical pain cases, such as trauma and acute cancer pain [2]. Hence, all patients should have access to acute pain service (APS) across all clinical specialties.

Historically, while some argue that APS was established in 1985 by a team of anesthesiologists [2], others posit that it started in 1988 as an anesthesiology-based postoperative pain management service. Guidelines pertaining to APS were first formally introduced in Australia in 1988 and thereafter in 1990 and 1992 in the United Kingdom and United States, respectively [3]. Between 1994 and 1996, there was a proliferation and formalization of APS, some reaching up to 77% in the big institutes in Australia and New Zealand, compared to 47% in the United Kingdom and 42% in the United States [2].

Since its establishment, anesthesiologists and other pain fellows have been shaping APS. However, despite significant progress, acute pain continues to be under or inadequately treated, particularly in African countries [4]. In addition, acute pain treatment and management have received insufficient attention, and inadequacies in postoperative pain relief have continued to be a major challenge. Kishore, Agarwal, and Gaur (2011) argue that acute pain patients may not be fit to receive standard pain management methods or may react to them later differently [5]. Furthermore, the ineffective treatment of acute pain can lead to chronic pain. This lack of adequate pain management affects 80% of the global population and is a serious problem in over 150 countries [6]. Baumhour et al. (2022) revealed challenges in implementing APS and bridging this gap, which include resource constraints and ineffective knowledge translation.

Currently, in Rwanda, the University Teaching Hospital of Butare formally lists a pain clinic service on its clinical service offerings [4, 7], yet there was no postoperative pain service reported between 2013 and 2017 [4, 8]. Like other countries in the region, implementing and accessing such a service in public hospitals remains a challenge [9]. Therefore, in line with this, this study aims to assess the knowledge, perceptions, and experiences of healthcare professionals about APS delivery at another tertiary-level teaching hospital in Rwanda, King Faisal Hospital Rwanda (KFH), with the aim to use these data to scale up APS delivery in Rwanda.

## 2. Materials and Methods

### 2.1. Study Design

This study employed a qualitative interpretive methodology aiming to explore the perceptions and experiences of healthcare professionals at KFH regarding APS delivery. The qualitative nature of this study was selected to enable an in-depth exploration of participants' perceptions and experiences regarding APS delivery at KFH, allowing for contextually specific findings and recommendations.

### 2.2. Study Setting

Data were collected at KFH, a tertiary-level teaching hospital with 160 beds and over 800 staff, located in Kigali, Rwanda. Currently, KFH employs one pain subspecialist, who is the only licensed pain subspecialist in the country and is overseeing the development of APS in the hospital. To date, progress includes the establishment of a pain outpatient service and the implementation of APS training and awareness sessions across all clinical departments. As KFH strengthens its specialized clinical care and teaching capacity, the integration of APS into both service and training and its scale-up across all clinical services is a hospital priority.

### 2.3. Data Collection

Data were collected via semistructured focus group discussions (FGDs) in April and May 2023, with each FGD having three to nine participants. Developed based on existing literature, the FGD tool was developed, piloted, and refined. It was developed in both English and Kinyarwanda and consisted of four thematic areas, including APS knowledge and experience; having standardized protocols; ways of introducing APS at KFH; and perceived barriers and mitigation strategies to implementing APS at KFH.

Forty-five study participants, consisting of 26 females (58%) and 19 males (42%), were selected through random stratified sampling to participate in the FGDs. These participants were divided into nine FGDs conducted across various units and departments at KFH, covering internal medicine, anesthesia and the operating theater, obstetrics and gynecology, the intensive care unit, pediatrics, accident and emergency, medical doctors, physiotherapy, and the surgical ward (Table 1).

### 2.4. Data Analysis

The FGDs were audio recorded, translated, and transcribed. The transcripts were coded inductively in Dedoose, with each transcript being coded by two researchers and then reviewed collectively by three researchers across all transcripts to ensure consistency in the coding. After the initial coding process, common themes and patterns were identified.

### 2.5. Reflexivity and Positionality

As an interpretive, qualitative study, the research team acknowledge their own influence over each stage of the research process, including data collection, analysis, and interpretation. To mitigate potential biases, reflexive discussions were held throughout the study to identify and address preconceptions related to APS delivery. Data analysis was conducted by multiple coders to incorporate diverse perspectives, and findings were regularly discussed within the team and validated with select participants to ensure accuracy and credibility. These steps were undertaken to uphold the validity of the study while recognizing the inherent subjectivity of qualitative research.

## 3. Results

Participants highlighted four key areas that may serve as either barriers or facilitators to implementing APS at KFH. These include healthcare provider skills and training; the development and implementation of standardized protocols; establishing a dedicated, interdisciplinary APS team; and patient awareness and education.

### 3.1. Healthcare Provider Skills and Training

Participants indicated that there is a current lack of skills regarding APS and that training healthcare providers would help mitigate this with the implementation of this service. Regarding a lack of existing skills, participants highlighted that this skills gap is both regarding general APS delivery and regarding existing protocols that may be in place:

*It's a barrier when you're trying to manage that kind of patient and yet you don't have the skills to manage their pain. Yes, I would like to have more knowledge about that.* (Physiotherapy FGD)

*The barriers we face include insufficient knowledge about APS and a lack of [knowledge of] protocols. While these are significant barriers, there are others that we can manage.* (Internal medicine FGD)

However, one participant noted that even if there are protocols in place, the current lack of healthcare provider skills will still be a challenge:

*The skills we have to deliver APS are insufficient. Of course, even if you have protocols and guidelines in place, you may find that they are not followed.* (Intensive care unit FGD)

Alongside the perceived skills gap highlighted by participants, they also indicated that integrating training for healthcare providers at KFH will help with APS delivery and mitigate this challenge:

*As the hospital takes care of the training of like, the ICU … the risk care provider, for Advanced Trauma Life Support (ATLS) or skin conductance responses (SCRs), [they] also need to put acute pain management. Every year to train new workers, they need to provide regular training.* (Medical doctors FGD)

A participant from the intensive care unit noted that the quality of training should also be considered to ensure that skills are acquired adequately:

*I also emphasize training. Sometimes institutions believe they have trained their staff. For example, during a 30 min in-service training session, when I sit for five minutes, I quickly return to the patient, and then I come back. It seems like this is not a training.* (Intensive care unit FGD)

Furthermore, one participant highlighted that training healthcare providers across different disciplines, especially nursing, would empower more staff to provide APS, without delaying patient care in the absence of a medical doctor:

*Sometimes you may receive a patient from the outpatient department or emergency, and the doctor may not be available because they are busy in the operating room performin surgery. In such cases, the nurse is not authorized to prescribe painkillers. As a result, the patient may experience a delay in receiving pain relief because the doctor is not available. But if many doctors and nurses are trained in pain management, if one doctor is not available, another may be able to prescribe it.* (Surgical FGD)

### 3.2. Development and Implementation of Standardized Protocols

Participants highlighted the benefits of having standardized protocols and policies in place as a facilitator to APS, noting that they establish a shared understanding among the team:

*We are trained differently, at different universities, and also work in different hospitals, so we have a difference of knowledge. When we combine it and put the same protocol, it is easy for everyone to treat the pain and help the other, who is not trained, to take it and read, to put it into practice.* (Medical doctors FGD)

Other participants emphasized that having standardized protocols provide consistency in patient care:

*It provides a way to manage pain in social settings. But if you have a protocol, you will find that you are managing patients well. It may indicate that when a patient arrives with a diagnosis of moderate pain, for instance, the initial treatment could involve paracetamol. However, without a protocol in place, a healthcare provider may choose to administer any drug of their preference.* (Accident and emergency FGD)

*When you have a protocol, everyone will handle the pain in the same way. So, there are no unnecessary changes in pain management.* (Internal medicine FGD)

Participants noted that there is currently a lack of awareness of APS protocols in place and that these should be either developed or communicated to the team:

*What I propose is that we implement a protocol that can be very helpful. If a protocol were established for this type of condition, it would apply to every condition we have and to every post-operative patient. And there is no cure for such a disease that causes pain. Implementing such protocols and emphasizing assessment: treating people as human beings.* (Pediatrics FGD)

One participant highlighted that the protocols should take into account APS for pediatric patients:

*In pediatrics, babies are unable to explain well where and how they feel. So, for example, the baby can cry when the father or mother is leaving. She wants to go with him, but she is unfamiliar with the hospital environment and is crying because of that. So, are you confused about whether to say that it is painful or if it is crying for that one? Other people can feel pain and choose to keep quiet. You can see the emotion in the expression. That is for the children.* (Pediatrics FGD)

### 3.3. Establishing a Dedicated Interdisciplinary Team

Not having a dedicated interdisciplinary team to address and manage APS was highlighted by participants as a barrier to its implementation, but it was also seen as a potential facilitator if established to oversee this service:

*In healthcare services, including in theatre, we require a dedicated pain management team to support both inpatient and outpatient care. This team is essential for providing independent pain management services.* (Anesthesia and operating theater FGD)

One participant further noted that the dedicated team could be responsible for training healthcare providers, thereby mitigating the need for increased skills in APS:

*In my opinion, there should be an official team responsible for training other healthcare professionals in pain management. This team could conduct regular training sessions in different departments to explain how to effectively manage pain…I believe that teams should consist of three to five members so that they can rotate throughout the entire hospital.* (Anesthesia and operating theatre FGD)

Furthermore, participants indicated that there is a critical need to integrate psychosocial support into APS, noting that pain not only is physical but also is the result of many factors:

*Due to the current system, there is a lack of emphasis on interdisciplinary and teamwork.*

*For example, meeting with a psychologist can greatly benefit the patient through talk therapy, addressing their psychosocial pain and leading to an improved sense of well-being.* (Physiotherapy FGD)

*I think most of the time we manage pain from injury or trauma here. But there is als psychological pain. I think we don't have enough skills to manage these patients. As others have said, we need to focus on managing the psychological effects of pain.* (Anesthesia and operating theater FGD)

### 3.4. Patient Awareness and Education

Participants indicated that they witness a general lack of patient awareness regarding APS and pain management in general. For example, one participant from the obstetrics and gynecology FGD highlighted this gap in the maternity ward:

*Here in maternity, it's like I don't know if they have the knowledge or understand how people perceive acute pain, like mothers in labor experiencing pain…She said, “No, I have to experience the pain.” So, you find the mother in severe pain due to labor, and she doesn' want to receive pain medication because she believes it will affect her ability to love her baby.* (Obstetrics and gynecology FGD)

Another participant corroborated this lack of awareness but also indicated that patients are not aware that pain management is a possibility in their clinical care and what their options are:

*Sometimes the patient is unaware of pain management. I am unsure if they can educate the patient about pain management. Sometimes patients want to receive morphine every time, which can be a barrier.* (Surgical FGD)

To mitigate the lack of patient awareness in APS, participants suggested that they can be educated both by KFH healthcare providers and on a broader level outside of the hospital.

*Outside of the hospital, I don't know if Rwandese people are aware that we have that service at KFH. Many people live with pain without knowing the reason behind it, and they are unaware of what they can do to ease their pain. They need to understand the cause of their pain and how to alleviate it.* (Intensive care unit FGD)

Another participant highlighted that patient education can also include topics such as addictions to pain medication and nonpharmacological approaches:

*Especially for patients who are addicted to medications such as morphine. Some patients come here and say, “I want this medication.” I believe they should receive an education.* (Accident & emergency FGD)

## 4. Discussion

To bridge the current gap in APS research in Rwanda and the region, this study provides contextually specific findings regarding barriers and facilitators to implementing APSs at KFH. The findings suggest that institutional systems, including standardized protocols, a dedicated team, and regular training opportunities, may help strengthen APS. Patient education and ensuring patients know their care options is another facilitator to improving APS.

### 4.1. Institutional Systems for APS

The findings suggest a need for standardized pain management protocols. The respondents concurred about the benefits of having these protocols in place, positing that they created a shared understanding among all practitioners regardless of their background and training [10]. To have uniformity in treatment and minimize variability in pain management techniques, standardized protocols for patient care are necessary. Such uniformity is very important, especially within a diverse institution such as KFH, since the different health professionals come from different training backgrounds and with varied approaches to pain management. This uniformity and shared understanding are supported by Corina et al., where they found that almost every staff stressed a very high need for a shared understanding of APS, its daily plan, and management [11]. In this study, there was a high need for a systematic approach to acute pain patients, including a comprehensive reporting system in the hospital [12]. The majority agreed that effective pain control improves patient recovery, and 88% believed that anesthetists should be involved in postoperative pain management. Overall, 85% of the respondents were satisfied with the APS [11].

Participants noted that without standardized protocols, there would be chances of making arbitrary treatment choices based on personal preferences rather than evidence-based practices. Implementing standardized protocols can guarantee equitable allocation of resources for optimal pain control to all patients thus enhancing general patient outcomes. In addition, clear guidelines can facilitate faster integration of new staff into the hospital's norms regarding how it handles pain. One systematic review of the evidence provided recommendations developed by a multidisciplinary expert panel on the management of postoperative pain, and they recommended standardized protocols which support the safety of the patient, provider, and the health system in general. This was also supported by the Australian and New Zealand College of Anesthetists recommendations in 2020, which emphasized that a holistic approach to an acute pain patient should be cared for with evidence-based care [13, 14].

### 4.2. Provisions for Healthcare Provider Training on Pain

Another vital point that was addressed is that healthcare staff need proper education on how to handle acute pain. They acknowledged a gap in APS delivery skills, sharing that most caregivers lack sufficient knowledge and skills to manage acute pain. This not only hampers the application of APS but also influences patient care. Such standard training is essential for effective service delivery, as it equips medical personnel with recent evidence-based practices in managing pain [14]. The training should be broad-based, encompassing doctors who are part of the team together with nurses and other health workers playing critical roles in patients. With this kind of training, which couples nonpharmacological strategies with the psychosocial dimensions of pain, medical personnel can better serve their patients in terms of holistic pain management [15, 16].

The participants also recommended the formation of an integrated, interdisciplinary APS team which would provide continual training and support across the hospital setting. Such a team could organize regular seminars and workshops to ensure that all employees are competent and familiarize themselves with APS protocols and procedures. Furthermore, continuous professional development opportunities can assist practitioners to keep up to date with developments in pain relief methods thereby improving their capacity for quality treatment through best practice interventional strategies. Core training containing pain education and involving every concerned staff (e.g., anesthesiologists, emergency physicians, nurses, and paramedical team) showed better results in acute pain management. The educational programs and seminars, along with standardized algorithms, should be developed [17].

### 4.3. Importance of Patient Education With Pain Management Options

This study also suggested levels of patient unawareness of APS and the lack of knowledge about pain management options. Participants perceived a general lack of understanding among patients regarding available pain management services, as well as their rights to access pain relief [18, 19]. This can result in poor pain management, since patients may not pursue or agree to treatments for relief from pain due to misunderstandings or other cultural factors [20]. For example, patients may refuse analgesics such as opioids during labor because they believe that it is important that they go through labor pain. It is therefore important that targeted patient educational interventions be developed, which challenge these myths. Informed consent empowers patients by educating them about what their choices are regarding pain management, including the advantages and disadvantages of different treatments [21]. Effective patient education should stretch beyond hospital boundaries into community-based awareness campaigns aimed at improving sensitization on various aspects of pain control [22]. Additionally, engaging with patients regarding their plans for managing their discomfort can allow for customization of interventions based on personal preferences and cultural considerations, thus promoting greater satisfaction with care [23].

### 4.4. Limitations and Implications for Future Research

One of the primary limitations of this study is the generalizability of the study. While its qualitative design provides in-depth insights into the perceptions and experiences of healthcare professionals, the findings may not be generalizable to other contexts or hospitals in Rwanda. Furthermore, the use of FGDs as the sole data collection method presents challenges such as groupthink, where participants may conform to dominant opinions, and the potential for certain voices to dominate the discussions, potentially skewing the results. Although efforts were made to mitigate these effects, these dynamics may still have influenced the findings. The absence of quantitative data, such as patient satisfaction scores or pain management outcomes, limits the ability to complement and validate the qualitative insights. Future studies could adopt a mixed-methods approach, incorporating both qualitative and quantitative data, as well as the perspectives of patients, to provide a more comprehensive and balanced perspective on APS implementation and its impact.

Further research should explore the long-term impacts of implementing standardized protocols and training programs on both healthcare provider performance and patient outcomes. Investigating the effectiveness of interdisciplinary teams in managing APS could also provide valuable insights into how collaborative approaches can enhance pain management services. Also, understanding patient awareness and education regarding pain management options is vital. Thus, future studies should focus on developing and evaluating educational interventions aimed at increasing patient knowledge and engagement in their pain management journey.

## 5. Conclusion

There is a critical need for the comprehensive strengthening of the delivery of APS globally. The findings of this study revealed that while there are existing barriers, such as inadequate training among healthcare providers and a lack of formalized interdisciplinary collaboration, there are also clear facilitators that can enhance APS delivery at KFH. Addressing these challenges is essential for ensuring that patients receive the comprehensive pain management they deserve. Regular opportunities for healthcare providers are also crucial. This investment in education will ultimately lead to improved patient outcomes and satisfaction. The establishment of a dedicated interdisciplinary APS team is another vital recommendation. By integrating various disciplines, including anesthesiology, nursing, and psychology, the team can address the multifaceted nature of pain and enhance the overall quality of care. Additionally, increasing patient awareness and education regarding pain management options is essential. Empowering patients with knowledge about their pain management choices can lead to better engagement in their care and improved satisfaction with the services provided. Therefore, by implementing standardized protocols, enhancing provider training, establishing a dedicated interdisciplinary team, and promoting patient education, KFH can significantly improve its pain management capabilities, thereby strengthening patient care.

## Figures and Tables

**Table 1:** Study participants by department and sex.

Department/units	Male	Female
ICU	1	2
Anesthesia and operating theater	3	3
Accident and emergency	2	7
Surgical ward	2	3
Internal medicine	0	4
Pediatrics	0	4
Physiotherapy	4	0
Gynecology	2	2
Medical doctor	5	1
*n*	19	26
Percentage	42%	58%

## Data Availability

The dataset and materials are available upon reasonable request to the corresponding author.

## Nomenclature

APS: Acute pain service
FGD: Focus group discussion
ICU: Intensive care unit
KFH: King Faisal Hospital Rwanda
